# Determinants of persistent smoking after acute myocardial infarction: an observational study

**DOI:** 10.1186/s12872-020-01641-8

**Published:** 2020-08-24

**Authors:** Jens Höpner, Udo Junge, Andrea Schmidt-Pokrzywniak, Christian Fischer, Rafael Mikolajczyk

**Affiliations:** Institute of Medical Epidemiology, Biometrics and Informatics, Martin-Luther University Halle-Wittenberg, Magdeburger Str. 8, 06112 Halle, Germany

**Keywords:** Smoking, Secondary prevention, Myocardial infarction, Predictors, Teachable moment, RHESA

## Abstract

**Background:**

Smoking cessation is one of the most effective secondary prevention measures after acute myocardial infarction (AMI). However, around 50% of smokers do not quit smoking after AMI. The aim of the present study is to estimate the proportion of patients quitting smoking and to identify determinants of persistent smoking after AMI in a region with increased cardiovascular mortality. We also assessed the time of smoking cessation after AMI.

**Methods:**

We used follow-up data of patients registered with the Regional Myocardial Infarction Registry in Saxony-Anhalt (RHESA) in Germany. We assessed smoking status and determinants of persistent smoking six weeks after discharge from hospital after AMI. Information on smoking, sociodemographic characteristics, risk factors for AMI, experienced symptoms of AMI, and clinical care were gathered in a computer-assisted telephone interview and questionnaires filled out by study subjects and physicians or study nurses.

**Results:**

Out of 372 smokers at the time of AMI, 191 (51.3%) reported that they quit smoking within six weeks after discharge from hospital after AMI. Strongest determinant of persistent smoking was a previous AMI before the current one (OR = 2.19, 95%CI 1.10–4.38) and strongest determinants of smoking cessation were experiencing complications in the hospital (0.37, 95%CI 0.12–1.12) and having a life partner (0.56, 95%CI 0.34–0.95). Most individuals who stopped smoking did so during the initial stay in the hospital, before the cardiac rehabilitation (CR).

**Conclusions:**

Persistent smoking after AMI and its determinants were similar in our region to previous studies. CR cannot be viewed as determinant of smoking cessation – more likely the same teachable moment induces behavioural change with regard to smoking and participation in CR.

## Background

For many years, Saxony-Anhalt has been one of the federal states in Germany with the highest incidence and mortality of acute myocardial infarction (AMI) [1]. In 2013, the age-standardized incidence of AMI (349 / 100,000 persons) was 28.3% above the national average. In order to identify reasons for the high incidence and mortality, the Regional Myocardial Infarction Registry (RHESA) was established in 2013 [2]. One potential explanation for the high incidence is the highest or second highest prevalence of common risk factors for AMI in Saxony-Anhalt among the German federal states (i.e. smoking, diabetes mellitus, arterial hypertension, obesity, increased waist circumference and the metabolic syndrome) [3]. However, it still remains unclear whether there is additional contribution of insufficient secondary prevention in Saxony-Anhalt including behavioral change, resulting in elevated rates of repeated infarctions with poor prognosis.

One of the most effective secondary prevention measures after AMI is smoking cessation [4]. A meta-analysis of twelve studies with a follow-up from two to ten years after AMI reported a 0.54 (95% CI 0.46–0.62) times reduced overall mortality for quitters compared to those who continued smoking [5]. However, according to the multi-centre study EUROASPIRE IV survey, 40–60% of AMI survivors in Europe do not stop smoking after AMI [6]. In Würzburg, the German study centre of that study, the quit rate was 47%.

One suggested mechanism to explain smoking cessation after AMI is that of a teachable moment - a naturally occurring event resulting in higher motivation for a change in risk behaviour [7]. Additionally, support provided at the right time can increase the possibility of a strong teachable moment encouraging smoking cessation [7, 8]. This support is available to patients during rehabilitation where, for example, the duration of the smoke-free policy that started in the hospital is prolonged, or pharmacological or psychological assistance is offered [9–11]. Predictors of persistent smoking after AMI have been identified in previous studies. These are sociodemographic characteristics [6, 12–15], smoking behaviour [11, 12], risk factors for AMI [12, 15], and the extent of medical interventions in the context of AMI [12, 14, 15]. Nevertheless, these factors can have different importance in various regions.

In the current analysis, we aimed to estimate the proportion of patients successful in quitting smoking after AMI in Saxony-Anhalt and to identify factors that determine persistent smoking after AMI in our region. We also address the question, when patients after AMI quit smoking in our population.

## Methods

### Study design and setting

RHESA-CARE 1 and 3 (RC1, RC3) are follow-up studies of patients who agreed to participate in the Regional Myocardial Infarction Registry of Saxony-Anhalt (RHESA) [2, 16]. The inclusion criteria for RHESA were age of 25 or more and being an inhabitant of the city of Halle (Saale) or the rural area of Altmark in Saxony-Anhalt (Germany) and being diagnosed with AMI in the hospital. While in RHESA we used multiple sources of information on mortality and morbidity of AMI in the population, in the follow-up studies only patients who provided informed consent during their stay in a hospital were included. In RC1, we contacted all AMI survivors who were registered with RHESA between April 2014 and January 2018 and agreed to participate in an active follow-up. The contact was six weeks after their hospital discharge. Since RHESA continued to recruit patients beyond the end of the RC1 study, we conducted an additional follow-up to include patients registered after January 2018, but included all patients registered with RHESA between June 2013 and January 2019. This follow up was conducted between February and May 2019 for all patients independently from discharge date after AMI including some participants of RC1. RC3 used a substantially shortened RC1 questionnaire with some additional questions for example regarding time of smoking cessation. Only those participants that reported in RC1 (or RC3 if RC1 information was not available) that they have been smokers at the time of AMI are part of this analysis.

### Variables

All variables of interest were gathered either through computer-assisted telephone interview (CATI) (RC1), written questionnaires (RC3) or by questionnaires answered by physicians or study nurses in the hospital (as part of RHESA). Quality and data comparability were ensured by using standardized and validated instruments that have been used in similar studies [17–19].

Smoking status at the time of AMI was self-reported in both, RC1 and RC3. In RC1 individuals were also asked about smoking status at the date of interview (six weeks after hospital discharge). In RC3, we asked if and when the individuals had stopped smoking and if and when they started smoking again. Thus, we were able to determine the smoking status six weeks after discharge from hospital also for those who participated only in RC3. Individuals that reported that they quit smoking within six weeks after discharge from hospital were considered as quitters. When continuation of daily or occasional smoking was reported six weeks after discharge from hospital, individuals were considered as persistent smokers. Based on a literature search, we identified four categories of potential determinants of smoking cessation: sociodemographic characteristics, risk factors for AMI, experienced symptoms of AMI, and clinical care.

For the first category of possible determinants of smoking cessation, we gathered information on age, sex, net income per household (metric variable assessed in steps of 500 €), education (according to the international standard classification of education (ISCED-97) with 3 levels (low, mid, high)), and having life partner. In the second category of potential determinants, we asked the participants for a confirmed diagnosis of arterial hypertension and diabetes mellitus and previous AMI. Participants were further asked if they had any intention to quit smoking before the AMI occurred. The third category of potential determinants included the presence of fear of death during the AMI as a binary variable. Information on the fourth category of potential determinants was gathered through hospital questionnaire answered by physicians or study nurses. The questionnaire contained information on the type of AMI (STEMI vs. NSTEMI) interventions after AMI (percutaneous coronary intervention (PCI)) or coronary artery bypass graft (CABG) vs. no intervention), complications in the hospital (i.e. shock, intubation, reanimation, severe bleeding, stroke or re-intervention, the number of newly prescribed drugs after hospital and the duration of hospitalisation (in days). We used the number of newly prescribed drugs as an auxiliary information for the perceived severity of heart disease. We assumed that this perceived severity can possibly strengthen the teachable moment associated with AMI and therefore increase the probability of smoking cessation.

In addition, we assessed the information on participation in cardiac rehabilitation (CR) within 2 weeks after hospital discharge in RC1 and in RC3, if it was not available in RC1. Using RC3 as a preferred source of information showed only minor differences in sensitivity analysis (data not shown).

### Statistical analysis

For sample characteristics, continuous variables were expressed as mean ± standard deviation for normally distributed data or as median and interquartile range for non-normally distributed data. Categorical variables were reported as frequencies and percentages (%).

Under the assumption of missing at random (MAR), we used multiple imputation to compensate incompleteness of information on age, sex, education, comorbidities (arterial hypertension, diabetes mellitus), fear of death during AMI, STEMI vs. non-STEMI status, intervention in the context of AMI, number of newly prescribed drugs, and duration of hospitalisation after AMI. Seventy complete data sets were generated according to the rounded-up percentage of incomplete cases. Fully conditional specification method was used in order to impute categorical variables and in order to not rely on normal distribution of continuous variables. The imputed models contained above-named variables, as well as information on region (Halle or Altmark) and on transfer between hospitals in order to conduct interventions after AMI (yes/ no). We used univariable logistic regression on the original dataset to obtain unadjusted effects of determinants on continuing smoking. We performed multivariable logistic regression on the imputed datasets and applied Rubin’s rule to combine their results [20]. Considering the high number of variables and to avoid overadjustment, we subdivided the covariates into 3 levels. Level 1 included information on the sociodemographic variables, level 2 information on the history of risk factors, and level 3 information on the symptoms of AMI and clinical care after AMI. Determinants where adjusted for all the variables in the same level and the previous level(s). All calculations were performed in SAS software version 9.4 (The SAS Institute, Cary, NC, USA).

## Results

### Sample characteristics

**Fig. 1:**
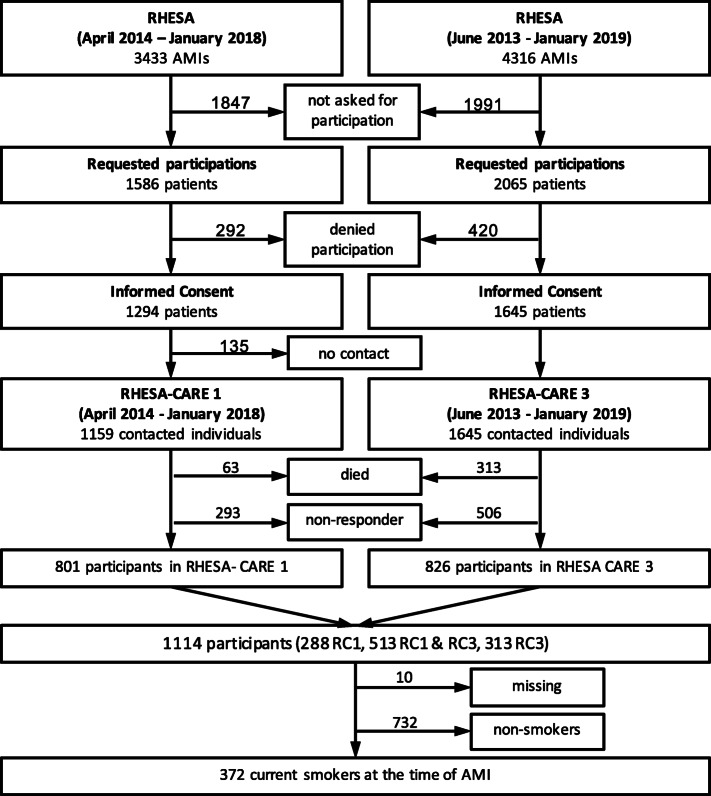
“Flow diagram of study population”

One thousand one hundred fourteen persons were enrolled in our study, of whom 288 only participated in RC1, 313 only in RC3, and 513 in both, RC1 and RC3 (Fig. 1). Three hundred seventy-two individuals reported being smokers at the time of AMI and about half of them (51.3%) reported having stopped smoking within six weeks after hospital discharge. Differences between persistent smokers and those who stopped smoking existed for sociodemographic characteristics, risk factors for AMI, and clinical care after AMI but not for the experienced AMI symptoms (Table 1). Respondents of RC3 had on average slightly higher mean age at the time of AMI (0.83 years), had more often male sex (71.8% vs. 69.7%), and more often were from the urban region city of Halle (58.0% vs. 56.2%) compared to respondents of RC1.

**Table 1:** Sample characteristics of persistent smokers and quitters

	persistent smokers (***N*** = 181)	quitters (***N*** = 191)	missing (%)
**Sociodemographic characteristics**			
Mean age^R^ in years (SD)	56.7 (10.2)	56.8 (8.9)	0.5
Male sex^R^, n (%)	125 (69.8)	140 (73.3)	0.5
Net income^1,3^ per household, n (%)			
0–1000 €	49 (28.2)	24 (13.3)	4.8
1001–3000 €	113 (64.9)	118 (65.6)	
> 3000 €	12 (6.9)	38 (21.1)	
Education^1^			
Low education	11 (8.5)	8 (5.7)	27.4
Intermediate education	90 (69.8)	89 (63.1)	
High education	28 (21.7)	44 (31.2)	
Life partner^1,3^, n (%)	99 (54.7)	145 (75.9)	0
**Risk factors for AMI**			
Arterial hypertension^R^, n (%)	143 (80.8)	139 (74.3)	2.2
Diabetes mellitus^R^, n (%)	45 (25.4)	37 (20.0)	2.7
Previous AMI^1,3^, n (%)	31 (17.1)	15 (7.9)	0
Intension to quit smoking^3^ n (%)	32 (28.8)	53 (37.1)	31.7
**Experienced symptoms of AMI**			
Fear of death^1^, n (%)	27 (21.8)	34 (25.4)	30.7
**Clinical care**			
STEMI^R^, n (%)	95 (53.1)	103 (53.9)	0.5
Intervention (PCI)^R^, n (%)	163 (97.0)	169 (96.6)	7.8
Intervention (CABG)^R^, n (%)	2 (1.2)	8 (4.6)	8.3
Complications^R^, n (%)	6 (3.4)	15 (7.9)	0.8
Mean number of new drugs^1^ (SD)	4.2 (2.2)	5.3 (2.0)	27.2
Median hospitalisation duration^R^ (Q1-Q3)	6 (4–7)	6 (5–8)	21.2
Attending cardiac rehabilitation^1,3^, n (%)	71 (39.4)	111 (58.4)	0.5

^R^ Information from questionnaire distributed in the hospitals (RHESA)

^1^ Information from computer assisted telephone interviews (RHESA-CARE 1)

^3^ Information from written questionnaires (RHESA-CARE 3)

### Determinants of smoking cessation

We identified having a previous AMI compared to no previous AMI (OR = 2.19, 95%CI 1.10–4.38) and receiving PCI or CABG compared to no intervention (OR = 1.53, 95%CI 0.66–3.54) as the strongest determinants of persistent smoking, and having a life partner compared to no life partner (OR = 0.56, 95%CI 0.34–0.95) and experiencing complications in the hospital compared to no complications (OR = 0.37, 95%CI 0.12–1.12) as the strongest determinants of smoking cessation (Table 2). Effects of age, sex, having STEMI and the duration of hospitalisation were close to the null effect.

**Table 2:** Determinants of persistent smoking from logistic regression models

	OR _**raw**_	OR _**Adjusted**_
***Sociodemographic characteristics***		
Age (per 5 years)	0.99	1.00 (0.98–1.02)
Sex (male vs. female)	0.84	1.07 (0.65–1.75)
Income (per 500 €)	0.76	0.82 (0.72–0.94)
Education (mid vs. low)	0.74	0.83 (0.32–2.18)
Education (high vs. low)	0.46	0.76 (0.26–2.19)
Life partner (yes vs. no)	0.38	0.56 (0.34–0.95)
***Risk factors for AMI***		
Hypertension (yes vs. no)	1.45	1.44 (0.84–2.49)
Diabetes mellitus (yes vs. no)	1.36	1.11 (0.64–1.94)
Previous AMI (yes vs. no)	2.42	2.19 (1.10–4.38)
Intension to quit smoking (yes vs. no)	0.69	0.83 (0.48–1.43)
***Symptoms***		
Fear of death (yes vs. no)	0.82	0.86 (0.45–1.65)
***Clinical Care***		
STEMI (yes vs. no)	0.97	1.07 (0.66–1.73)
Intervention (yes vs. no)	1.14	1.53 (0.66–3.54)
Complication (yes vs. no)	0.40	0.37 (0.12–1.12)
New prescribed drugs (per 1 drug)	0.79	0.86 (0.75–0.98)
Hospitalisation duration (per 3 days)	0.79	0.95 (0.87–1.02)

### Time of smoking cessation

Among those who stopped smoking within six weeks after hospital discharge, most stopped smoking already during hospital stay (Table 3). Among those who attended CR, some stopped smoking before CR. Overall, smoking cessation was more common and restarting smoking less common among those who attended CR in comparison to those who did not, but the attendance to CR cannot be considered as the cause of smoking cessation, as it occurred mostly before CR.

**Table 3:** Time of smoking cessation

		CR participants (***N*** = 133)		Non-CR-participants (***N*** = 119)	
Time of quitting smoking after AMI		quitter^b^ n (%)	Backslider^c^ n (%)	n (%) quitter (incl. temporary)	n (%) backslider
During hospitalisation		69 (51.9)	4 (5.8)	50 (42.0)	12 (24.0)
within 6 weeks after hospital discharge	before CR^a^	4 (3.0)	3 (75.0)	12 (10.1)	7 (58.3)
	during CR^a^	7 (5.3)	3 (42.9)		

*CR* cardiac rehabilitation

^a^ only for those who attended CR

^b^ those who did not smoke six weeks after discharge

^c^ among the quitter those who restarted smoking after six weeks

## Discussion

In our study population, about 50% of smoking AMI patients stopped smoking within six weeks after discharge from hospital after AMI. The strongest determinants of smoking cessation were having a life partner and having experienced complications in the hospital. Persistent smoking was most strongly determined by having previous myocardial infarction and by receiving PCI or CABG. More than 80% of those who stopped smoking after AMI did so before hospital discharge and thus also before CR.

### Implications and comparison to previous studies

The proportion of quitters in our study population corresponded to the average quit rate of European patients with coronary heart disease [6]. The proportion of quitters in our study population was even slightly higher than the proportion reported for Germany in the above study (51% vs 47%). Thus, it does not appear that this factor could explain the morbidity/mortality excess in our region in Germany.

Factors that were associated with smoking cessation were found in the category of sociodemographic characteristics, risk factors for AMI, and clinical care, but not in symptoms of AMI. We found no association between age at hospitalisation due to AMI and smoking cessation. Most of the previous studies showed similar results [13–15], although some studies showed a positive association between higher age and smoking cessation [6, 12]. Furthermore, we saw no association between sex and smoking cessation. While one other study showed a similar finding [14], some other studies showed a protective effect [6, 13, 15] and one other a harmful effect [12] of male sex on continuous smoking in comparison to female sex. There can be various explanations of these findings – including different smoking behaviors of the respective populations and the different survey periods. A higher net household income had a positive effect on smoking cessation which is concordant to other studies [12, 21]. The German Health Interview and Examination Survey for Adults (DEGS1) from 2013 showed that higher socio economic status is not only associated with lower prevalence of smoking, but also with higher cessation independently of AMI [22]. Having a life partner was associated more often with smoking cessation compared to not having a life partner, which has also been shown in previous studies [12, 14, 15]. We hypothesise that people having a life partner feel not only responsible for themselves, they might feel a responsibility for their partner as well. This may increase the emotional component of the teachable moment and could lead to a higher cessation rate. Additionally, those people may experience additional support in the process of quitting and staying away from cigarette smoking. Individuals diagnosed with arterial hypertension had a higher risk to continue smoking. This effect was also found elsewhere [12]. We found a strong association between having a history of AMI and continuous smoking. People that are not able to stop smoking after their first myocardial infarction are less likely to stop smoking after their next cardiac events. A comparison with other studies is not feasible because those either excluded subjects with previous myocardial infarctions in the first place, or they only provided descriptive data about cessation rates [12, 15]. In our study, we found that subjects who suffered complications in the hospital had the highest chance of quitting smoking. Since only a few people in the hospital suffered complications, the confidence interval is very wide. A longer stay in hospital and a higher number of newly prescribed drugs after AMI were only slightly associated with smoking cessation. The number of new drugs was used as a proxy for the perceived severity of heart disease. We can imagine that taking new drugs every day will give patients a lasting feeling of illness. In contrast to other studies [12, 14], we found a positive association between being subject of a PCI or CABG on the one side and continuing smoking on the other. However, more than 90% of the subjects in our study got a PCI, and thus the non-intervention group was very small. Nevertheless, many of the cited publications looking for predictors of smoking cessation have been published over 20 years ago. It could be interpreted as a signal suggesting that today’s hospital interventions and the stay itself are no longer considered as life-threatening as it was years before.

Several previous studies assessed CR as potential determinant of smoking cessation [11, 13, 23, 24]. We could show in our study, that those who participated in CR not only were more likely to quit smoking, they mostly quit already before attending CR. This may suggest that those who attend CR are more strongly motivated to modify their risk behavior even before attending CR compared to those who do not attend CR. Those who attend CR are also more likely not to restart smoking – but we cannot say if this is also due to selection, or possibly because of additional support during CR.

### Strength and limitations

The strength of our study is the use of data from a population-based registry of AMI with a systematic follow up – in contrast to studies based on hospital samples only. Furthermore, we were able to assess a very broad set of determinants of smoking cessation after AMI.

At the same time, our study has some limitations. Information on smoking and several of the variables assessed as determinants was self-reported and thus subject to reporting bias. The recall bias could be increased by the fact that for some participants the AMI was several years ago. We had relatively limited information on smoking behavior before AMI. Furthermore, we were not able to shed light on whether CR contributes to smoking cessation – we could only show that it would be incorrect to attribute smoking cessation to CR. Although the sample size is relatively small, we could answer the posed questions. In regression analyses, the sample fulfilled the rule of thumb with at least 10 events per variable [25].

## Conclusion

In our cohort of AMI patients from a population-based registry in a region with a comparably high cardiovascular morbidity and mortality, 51.3% stopped smoking within six weeks after discharge from hospital, similarly to other European and German data. Also, the determinants of smoking cessation were similar to previous studies, therefore an explanation of elevated mortality has to be searched in other areas. While CR was considered determinant of smoking cessation in previous studies, it appears that among those who attend CR, most stop smoking already before starting CR.

## Data Availability

The datasets used and/or analysed during the current study are available from the corresponding author on request.

## Abbreviations

AMI: Acute myocardial infarction
CABG: Coronary artery bypass graft
CATI: Computer assisted telephone interview
CR: Cardiac rehabilitation
PCI: Percutaneous coronary intervention
RC1: RHESA-care 1
RC3: RHESA-care 3
RHESA: Regional myocardial infarction registry
STEMI: ST-elevation myocardial infarction
